# RUNX1 knockdown induced apoptosis and impaired EMT in high-grade serous ovarian cancer cells

**DOI:** 10.1186/s12967-023-04762-8

**Published:** 2023-12-06

**Authors:** Yuanzhi Chen, Zhicheng He, Shuting Yang, Cheng Chen, Wenyong Xiong, YingYing He, Shubai Liu

**Affiliations:** 1 State Key Laboratory of Phytochemistry and Plant Resources in West China, Kunming Institute of Botany, Chinese Academy of Sciences, #132 Lanhei Road, Panlong District, Kunming, 650201 Yunnan People’s Republic of China; 2 University of Chinese Academy of Sciences, Beijing, 100049 China; 3 School of Chemical Science & Technology, Yunnan University, Kunming, 650091 Yunnan China; 4 School of Life Science, Yunnan University, Kunming, China

**Keywords:** Runt-related transcription factor 1 (RUNX1), High-grade serous ovarian carcinoma (HGSOC), Apoptosis, EMT, Clinical therapeutic target

## Abstract

Ovarian cancer is the leading cause of death from gynecologic illnesses worldwide. High-grade serous ovarian cancer (HGSOC) is a gynecological tumor that accounts for roughly 70% of ovarian cancer deaths in women. Runt-related transcription factor 1(RUNX1) proteins were identified with overexpression in the HGSOC. However, the roles of RUNX1 in the development of HGSOC are poorly understood. In this study, combined with whole-transcriptome analysis and multiple research methods, RUNX1 was identified as vital in developing HGSOC. RUNX1 knockdown inhibits the physiological function of ovarian cancer cells and regulates apoptosis through the FOXO1-Bcl2 axis. Down-regulated RUNX1 impairs EMT function through the EGFR-AKT-STAT3 axis signaling. In addition, RUNX1 knockdown can significantly increase the sensitivity to clinical drug therapy for ovarian cancer. It is strongly suggested that RUNX1 work as a potential diagnostic and therapeutic target for HGSOC patients with better prognoses and treatment options. It is possible to generate novel potential targeted therapy strategies and translational applications for serous ovarian carcinoma patients with better clinical outcomes.

**Supplementary Information:**

The online version contains supplementary material available at 10.1186/s12967-023-04762-8.

## Introduction

Ovarian cancer is one of the most difficult malignant tumors of the female reproductive system and is responsible for more than 140,000 deaths in women worldwide each year [1]. Nearly 314,000 women were estimated to be diagnosed with ovarian cancer, and 207,000 died from the disease in 2020, varying rates worldwide. High-grade serous ovarian carcinoma (HGSOC) is estimated to account for 50–60% of all ovarian malignancies [2, 3]. Clinically, patients with HGSOC usually present in the advanced stage, and the 5 year survival rate is generally less than 30% [4]. It is essential to explore the mechanism, clarify the pathogenesis of HGSOC, search for potential tumor markers or therapeutic targets, and develop new therapeutic strategies to generate better outcomes for patients.

Runt-related transcription factor 1 (RUNX1), a member of the RUNX family that includes RUNX2 and RUNX3, is named for its common Runt-like DNA binding domain [5] and works as an important transcription factor in histogenesis [6]. RUNX1 regulates hematopoietic stem cells and the hematopoietic process [7]. RUNX1 was first identified in cancer by highly correlating the pathogenesis of leukemia disease with RUNX1 gene chromosome heterotopic and mutations [8]. RUNX1 indicates particular tissue specificity and does either inhibit or promote substantive malignant tumors derived from different tissue sources [9, 10]. RUNX1 plays a crucial, pivotal role in many cancer types, raising concerns about RUNX1 as a biomarker for cancer. RUNX1 showed lower expression in breast tumor tissues than normal tissues and sharply company with the tumor malignancy increase [11]. On the contrary, RUNX1 could promote proliferation in glioblastoma by activating the MAPK signaling pathway, thus promoting tumor invasion, metastasis and angiogenesis [12]. RUNX1 expression in ovarian cancer cells has been found to promote the proliferation and migration of ovarian cancer tumor cells [13]. However, it is still elusive for the mechanism of RUNX1 in HGSOC.

In this study, the HGSOC samples in TCGA were employed to evaluate the potential of RUNX1 as a biomarker for diagnosing and treating target. SKOV3 and OVCAR3 cell lines was used to investigate the role of RUNX1 in HGSOC by whole transcription profile analysis and multiple functional research methods. The physiological functions were evaluated in RUNX1 knockdown ovarian cancer cells, such as proliferation, migration, and invasion. The impacted signaling pathways by RUNX1 were analysed and enriched for key cellular functions. Finally, it was evaluated that the synergic effect of RUNX1 deletion enhanced the sensitivity of HGSOC cells to classic clinical drugs.

## Materials and methods

### Plasmids and transfection

The GeneChem company has created and constructed short-hairpin RNA (shRNA) targeting RUNX1 (shRUNX1) and control shRNA (mock) (Shanghai, China). The corresponding shRNA sequences were listed (Additional file 1: Table S1). Transfections were carried out according to the Lipofectamine 2000 (Invitrogen, USA) protocol. RUNX1 knockdown in SKOV3 and OVCAR3 cells were confirmed by Western Blotting analysis after two weeks of screening in media with 2.0 μg/ml puromycin (Sigma-Aldrich Company, USA). This method was used in subsequent experiments to stabilize cell lines.

### RUNX1 gene expression and survival curves

The RUNX1 genes were explored in the Cancer Genomics dataset (TCGA) through GEPIA and investigation of genetic alterations associated with the cases of patients with high grade serous ovarian cancer. The Kaplan–Meier plots were generated from an online data set (http://www.kmplot.com). Overall survival (OS) analysis was performed using patient information. The patient population was divided by the median value.

### Cell culture

Human high-grade serous ovarian carcinoma cell lines (SKOV3, OVCAR3) and ovarian epithelial cells (IOSE80) were purchased from American Type Culture Collection. SKOV3 and IOSE80 are cultured in 1640 medium, supplemented with 10% fetal bovine serum (Gibco, USA) at 37 °C in a humidified 5% CO_2_ atmosphere. OVCAR3 is cultured in 1640 medium containing 1% insulin. Moreover, 10% fetal bovine serum (Gibco, USA) was supplemented with 37 °C water saturated with 5% CO_2_ atmosphere.

### Cell Transwell migration and invasion assays

Matrigel invasion assay was performed using 24-well transwells (8-μm pore size, Corning, USA) precoated with Matrigel. Isolated cells (1 × 10^5^) were suspended in 200 μL non-FBS cell culture medium was added to the upper chamber, and 600 μL culture medium containing 20% FBS was added to the lower chamber and cultured at 37 °C. After the specified time, cells migrated to the lower surface of the membrane were fixed with 4% paraformaldehyde for 15 min and stained with crystal violet for 20 min. The membrane was air-dried after further washing with PBS, and the number of cells on the membrane was calculated under an optical microscope at × 200 magnification. The cells numbers were calculated by using Image J software. The number of migrated cells was expressed as the average of five randomly selected fields.

### Colony-forming assays in agar gel

The scramble control and stable RUNX1 KD Cells (SKOV3, OVCAR3) were cultured in soft agar gel for additional 15 day cultured followed the protocol. The cancer cells formed colonies were stained (0.5% crystal violet/20% ethanol) and taken image by light microscope. The colonies numbers were calculated by using Image J software.

### Cell proliferation

Cell proliferation was evaluated by Cell Counting Kit-8 (Abcam, UK). The cells were inoculated on 96-well plates at a density of 5 × 10^3^ cells per well. After the experimental treatment, CCK-8 (10 μl/well) was added to the wells at the specified time point. After incubation for two hours, the reaction products were measured at 450 nm.

### Western blot

After treated for a specific time, the cells were gently washed three times with PBS and then cleaved in RIPA lysis buffer (Beyotime Biotechnology, China). The Beyotime assay kit was used to determine the protein concentration of cell extracts, which was used to adjust the protein concentration in the experiment. SDS-PAGE isolated the proteins on 8%, 10%, 12%, or 15% gel at 12 μl/well and wet-transferred to polyvinylidene difluoride (PVDF) membranes (Millipore, USA). The membrane was enclosed at room temperature for one hour using 5% skim milk dissolved in TBST solution (FD bioscience, China), and incubated with a specific protein primary antibody at 4 °C overnight. The membranes were washed three times in TBS-T solution for 10 min each time and set with either goat anti-rabbit (1:3000, Abcam, UK) or goat anti-mouse (1:5000, Abcam, UK) secondary antibodies at room temperature for one h. Immunoreactive proteins were visualized according to the manufacturer's instructions before exposure of PVDF membranes using Tanon 5200 (Tanon, China) using an enhanced chemiluminescence kit (Thermo Fisher Scientific, USA). The standard methods using the following antibodies: RUNX1 (Cusabio, China), p-FOXO1^Ser256^ (CST, USA), FOXO1 (CST, USA), Bcl-2 (Proteintech, China), caspase-3 (CST, USA), BAX (Proteintech, China), N-Cadherin (Proteintech, China), E-Cadherin (Proteintech, China), Vimentin (Proteintech, China), SNAIL (Proteintech, China), p-STAT3^Tyr705^ (CST, USA), STAT3 (CST, USA), p-EGFR^Tyr1068^ (CST, USA), EGFR (CST, USA), p-AKT^Ser473^(CST, USA), p-AKT^The308^ (CST, USA), AKT(Proteintech, China), β-Actin (Proteintech, China), GAPDH (Proteintech, China).

### Whole transcript expression profiling analysis

RNA was extracted from SKOV3 shRNA control and RUNX1 knockdown cell lines using TRIzol reagent (Invitrogen, Carlsbad, CA). The quality and quantity of the RNA were tested using spectrophotometric analysis and Bioanalyzer (Agilent Technologies, Santa Clara, CA). RNA was extracted from cell lines using TRIzol reagent (Invitrogen, Carlsbad, CA). 1 ug of RNA per each sample were used for target labeling by a two-round amplification protocol. Expression profiles were determined using Affymetrix 1.1 Human Gene ST arrays according to the manufacturer's instructions. For RNA expression profiling, each sample toke 4.5 μg of fragmented, labeled and hybridized with per GeneChip (Human Gene whole transcript 1.1 ST Arrays, Affymetrix) were processed on the Affymetrix GeneAtlas Fluidic station. The data were normalized to the median of all probe sets on each chip and lastly by “per gene”. For example, normalized to the median expression of each probe set across all samples. Expression data were normalized, background-corrected, and log2-transformed for parametric analysis. All Affymetrix control genes were removed and the remaining Affymetrix probe clusters were imported into the Affymetrix transcription analysis console. Significantly Differentiated expression genes were identified using significance analysis of microarrays (SAM) with the R package ‘samr’ (false discovery rate (FDR) < 0.05; fold change > 2) and determining the gene list based on the number of significant genes that were identified by fold change. Two-dimensional hierarchical clusters are generated. Gene significantly enrichment analysis (GSEA) were applied to identify enrichment functions, pathway and networks.

### Metascape pathway analysis

Gene ontology (GO) and pathway enrichment analysis of RUNX1 KD-associated significantly changed genes were performed using Metascape [14]. In this study, an ordered list of genes was first generated by GSEA based on correlation with RUNX1 KD. The significant survival difference observed between the control, and RUNX1 KD was elucidated. Gene set permutations were performed 1000 times for each analysis. The nominal *p*-value and normalized enrichment score (NES) were used to classify the pathways enriched in each phenotype.

### Immunofluorescence and immunohistochemistry

Stable knockdown cells were seeded on a six-well plate with a pre-placed lid for immunofluorescence. The cells were cultured for 48 h, fixed at room temperature with 4% formaldehyde for 10 min, and washed with washing buffer solution (0.02% Tween 20/PBS) 3 times. Then, the cells were infiltrated at room temperature with 0.5% Triton X-100/ PBS for 10 min. The cells were washed three times (5 min each) with a washing buffer, then incubated at room temperature for 30 min with 1.5% bovine serum albumin (BSA)/phosphate-buffered saline (PBS) solution (closed solution). E-cadherin antibody (Proteintech, China), Snail antibody (Proteintech, China), and Vimentin antibody (Proteintech, China) were incubated overnight in a sealed solution at 4 ℃. The fluorescent secondary antibody of goat against rabbit was incubated at 4 ℃ for 1–2 h and then backed with DAPI (Thermo Fisher, USA). The samples were mounted with an anti-quenching reagent (Life Technologies, USA) and imaged on confocal microscope.

Ovarian cancer tumor tissue microarray was bought from bioaitech (product ID: F801401, Xi’an, China), which contained normal and cancerous ovarian tissues with definite pathological diagnoses. The microarray includes 80 ovaries of surface epithelial origin and malignant tumor specimens representing five distinct histological types. We used this microarray to detect RUNX1 expression in ovarian tumor tissue. Briefly, the slide was dewaxed in xylene twice for 15 min each time and rehydrated with a series of graded alcohol buffers for about 20 min. Endogenous peroxidase was blocked by treatment with 3% catalase for 10 min, then boiled in a pressure cooker for 3 min to recover the antigens. Sections were incubated overnight with RUNX1 antibody (Cusabio, China) at 4 ℃. Incubation was performed at 37 °C with the secondary antibody sheep anti-Rabbit IgG-Biotin for 30 min, performed the chromogenic was performed with a DAB Substrate Kit, then counterstained with hematoxylin. The slides were then dehydrated in a graded alcohol buffer and covered with cover slides. The staining intensity and percentage of RUNX1-positive tumor cells were observed under a microscope. ImageJ software and IHC Profiler plug-in [15] were used to monitor and evaluate the tumor tissue images after staining. Slides were counted and then automatically rated for immunohistochemical strength. IHC score values were expressed as mean ± SEM. The ANOVA analysis compared the mean values of IHC scores between benign and different tumor histological types.

### Flow cytometry analysis

Samples were measured AV-AF647/PI through flow cytometry for cell apoptosis analysis. Cells were stained according to the method recommended by the manufacturer of the apoptosis kit (Yeasen, China). In brief, cells from the stable knockdown and control cell lines in this study (5–10 × 10^4^) were spread in six-well plates and cultured for 24 h. Cells were washed with PBS, collected with trypsin solution, and washed three times with PBS. AV-AF647/PI was added to the tubes and gently mixed with the cells for 10 min at room temperature in the dark. Stained cells were washed three times with cold PBS, fixed, and permeabilized with 0.5% Triton X-100 in PBS for 5 min. Finally, cells were analysed by flow cytometry, and data were collected for results analysis.

### Correlation analysis between RUNX1 expression pattern and clinical medication

The RUNX1 expression profiles data from ROC plotter (http://www.rocplot.org/crc/index), which can link RUNX1 expression and response to therapy using transcriptome-level data, were used to investigate the clinical prognosis of RUNX1 and the relationship between RUNX1 expression levels and six clinical drug treatments for ovarian cancer. The correspond AUC of RUNX1 and drugs also were examined. The influence for cell proliferation after paclitaxel, taxane, and platinum agents (cisplatin) treated was measured by cell proliferation assay as shown in part “Cell proliferation”.

### Statistical analysis

Data are presented as Means ± SEM. The significance of differences for the experimental values were compared using the student t-test through Prism software (GraphPad Software, Inc., San Diego, CA, USA). *P-value* less than 0.05 was defined as a significant difference (*p < 0.05; **p < 0.01; ***p < 0.001).

## Results

### RUNX1 over-expressed and correlated with patient’s poor prognosis

**Fig. 1:**
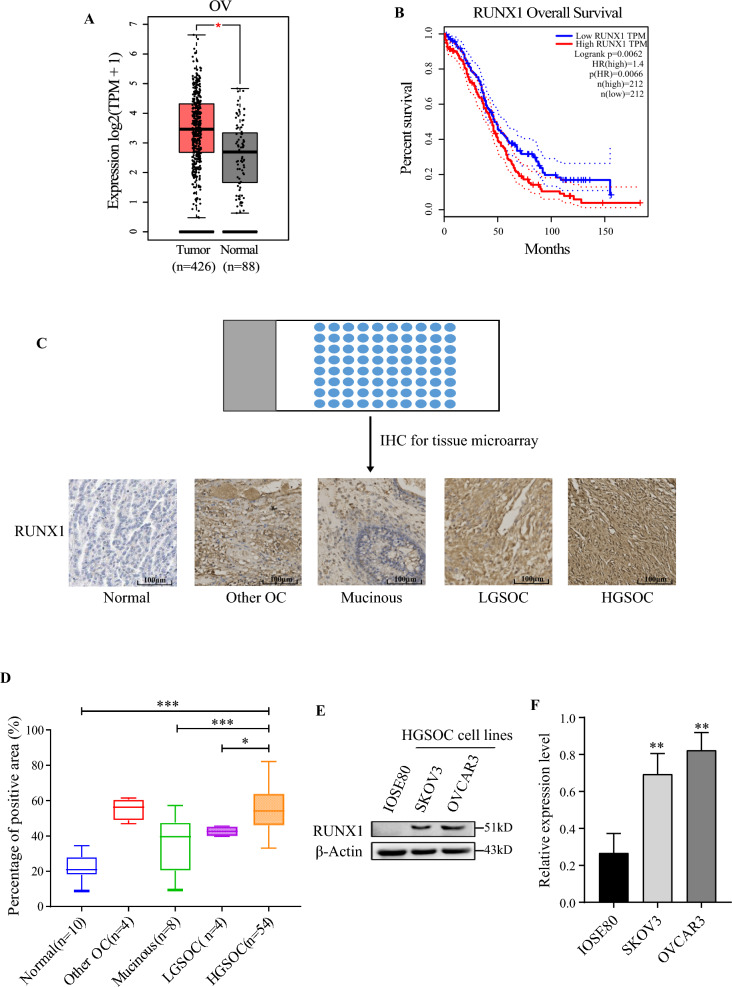
The expression comparison of RUNX1 in ovarian cancer. The expression pattern of RUNX1 in ovarian tumor tissues and NT (normal tissues) (**A**), including 426 tumor tissues (TCGA) and 88 normal tissues (GTEx). The comparison of the expression level for RUNX1 mRNA was performed. OS (*p* = 0.0062, **B**) of ovarian cancer patients was significantly positively associated with the expression of RUNX1. Representative images are shown the expression of RUNX1 in normal ovarian tissues, mucinous ovarian cancer tissues, LGSOC (low grade serous ovarian cancer) tissues, HGSOC (high grade serous ovarian cancer) tissues and other types of ovarian cancer tissues (**C**). The IHC staining scores were statistically analyzed between normal and ovarian cancer tissues (**D**). The overexpression of RUNX1 was analyzed by western blot in normal ovarian epithelial cells and HGSOC cell lines (**E** and **F**)

Blast in the GEPIA Cancer Genome database, RUNX1 was found to be overexpressed in 426 ovarian tumor tissues compared with normal ovarian tissues (n = 88, Fig. 1A). Meanwhile, patients with high RUNX1 expression had a significantly shorter survival time than those with low RUNX1 expression (Fig. 1B). HGSOC patients demonstrated about 3% genetic alternation in RUNX1 gene, including mutation, amplification, and deep deletion (Additional file 1: Fig. S1A). While patients with genetic alternation of RUNX1 were significantly extended the overall survival and disease-free survival time (Additional file 1: Fig. S1B–C). Based on the IHC staining results of ovarian tumor tissues microarray, the expression level of RUNX1 was significantly higher in high-grade serous ovarian cancer (HGSOC) tissues compared to low-grade serous ovarian cancer (LGSOC) tissues, mucinous ovarian cancer tissues, while its expression remained normal in non-cancerous ovarian tissues (Fig. 1C–D). Western blot analysis revealed RUNX1 overexpression in high-grade serous ovarian cancer cell lines (SKOV3 and OVCAR3), compared to human normal ovarian epithelial cells (IOSE80) (Fig. 1E–F). Therefore, these two ovarian cancer cell lines were selected for constructing RUNX1 stable knockdown cell lines and subsequent experiments. Taken together, the over-expression of RUNX1 in HGSOC suggest that RUNX1 may play an essential role in the progression of ovarian cancer and seriously affect the prognosis of ovarian cancer patients.

### RUNX1 knockdown impaired the cellular functions of ovarian cancer cells

**Fig. 2:**
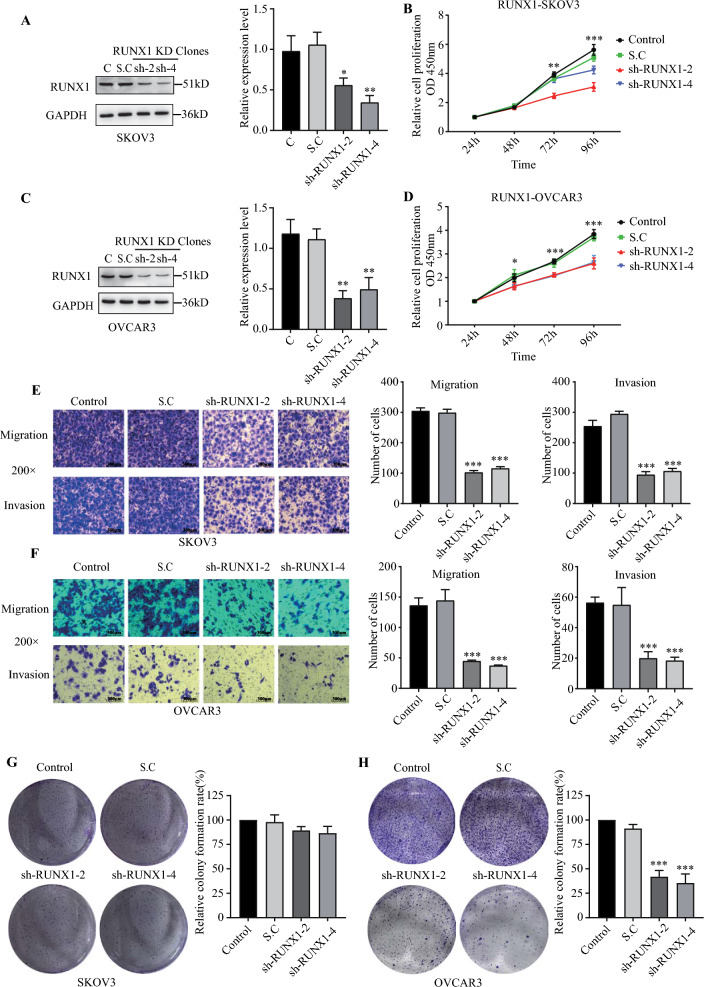
The RUNX1 mediated the abnormal proliferation, invasion, and migration in the ovarian cancer cells. Western blot confirmed RUNX1 stable knockdown in SKOV3 cells constructed by plasmids containing RUNX1-targeting shRNA (**A**). CCK8 assay showed that knockdown of the expression levels of RUNX1 reduced the proliferation ability of SKOV3 (**B**). Western blot confirmed RUNX1 stable knockdown in OVCAR3 cells constructed by plasmids containing RUNX1-targeting shRNA (**C**). CCK8 assay showed that knockdown of the expression levels of RUNX1 reduced the proliferation ability of OVCAR3 (**D**). Differences in the migration and invasion ability of cells after the knockdown of RUNX1 expression levels and the migration and invasion ability of the cells were decreased when RUNX1 was knocked down (**E** and **F**, 200 ×). Colony formation assay showed that knockdown of the expression levels of RUNX1 reduced the ability of colony formation of SKOV3 and OVCAR3 (**G** and **H**). A representative experimental result was generated from three independent experiments. *p < 0.05, **p < 0.01, and ***p < 0.001 compared to control cells expressing a scramble shRNA control, paired t-test

Two ovarian cancer cell lines (SKOV3, OVCAR3) were transfected with the constructed RUNX1 shRNA to establish stable RUNX1 knockdown cell line. Western blot results indicated that RUNX1 protein expression was significantly decreased in two RUNX1 knockdown constructs (sh-2, sh-4) compared with wild-type cell line (control) and blank vector transfected cell line (Scramble Control, S.C) (Fig. 2A, C, Additional file 1: Fig. S2A–B). RUNX1 knockdown resulted in the proliferation, invasion and migration abilities of cancer cell lines (SKOV3, OVCAR3) significantly reduced than control cell lines (Fig. 2B, D–F). The capacity of forming colonies in vitro was no significant difference in RUNX1-KD SKOV3 cells than control cells (Fig. 2G). However, there was significantly reduced in RUNX1-KD than control in OVCAR3 cells (Fig. 2H). These results indicated that down-regulated RUNX1 greatly affected the proliferation of high grade serous ovarian cancer cells.

### Key pathways and critical functions regulated by RUNX1 in ovarian cancer cells

**Fig. 3:**
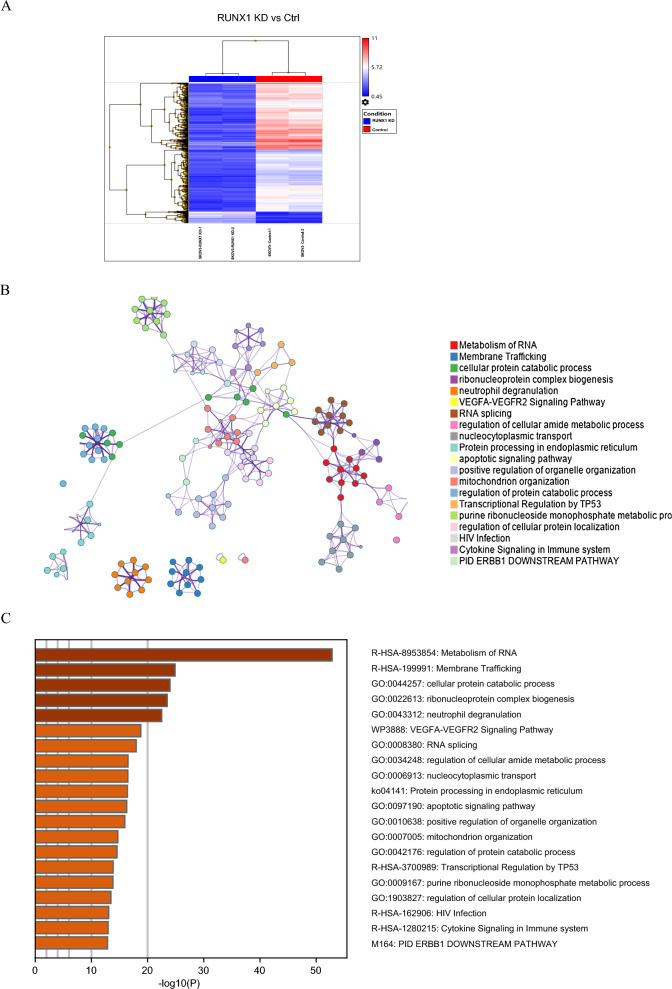
Functional and pathway enrichment analysis of the RUNX1 knockdown expression profiling in ovarian cancer cells. Heat map demonstrated the significantly changed genes hierarchical cluster analysis of RUNX1 knockdown transcription profiles in SKOV3 ovarian cancer cells. The significantly changed genes of RUNX1 vs control were screened and identified (**A**). Representative Molecular Complex Detection (MCODE) network node demonstrated the connection of significantly changed genes regulated by RUNX1 knockdown (**B**). Representative Molecular clusters were enriched. Left panel, heatmap of the top 20 enriched terms (**C**). Metascape analysis revealed a Network of enriched sets colored by ID. Threshold value: 0.3 kappa score; similarity score > 0.3. b Heatmap colored arranged by *p* -values

Whole transcription profiles analysis was performed to identify the significant regulated genes between RUNX1 knock-down and control cells. Filtered with *p-value* (p < 0.001) and fold change (RUNX1 knock-down vs control cells, FC > 2.0 or FC < 0.50), RUNX1 knockdown led to the upregulation of 1396 genes and downregulation of 42 genes after data normalization and significance analysis. These genes were identified as significantly altered and computationally aggregated in RUNX1 KD versus control SKOV3 cells (Fig. 3A). In addition, molecular Complex Detection (MCODE) network node demonstrated the connection of significantly changed genes regulated by RUNX1 knockdown (Fig. 3B). The top 20 enrichment signaling pathways were summarized, including the apoptotic signaling pathway, VEGFA-VEGFR2 Signaling Pathway, and transcriptional regulation by TP53, etc. (Fig. 3C). In particular, the apoptotic signaling pathway was highlighted in the RUNX1 KD cancer cells’ enrichment pathways and chosen for further investigation. Together, RUNX1 may play an essential regulatory role in ovarian cancer development.

### Knockdown RUNX1 activated apoptosis through the FOXO1-Bcl2 axis

**Fig. 4:**
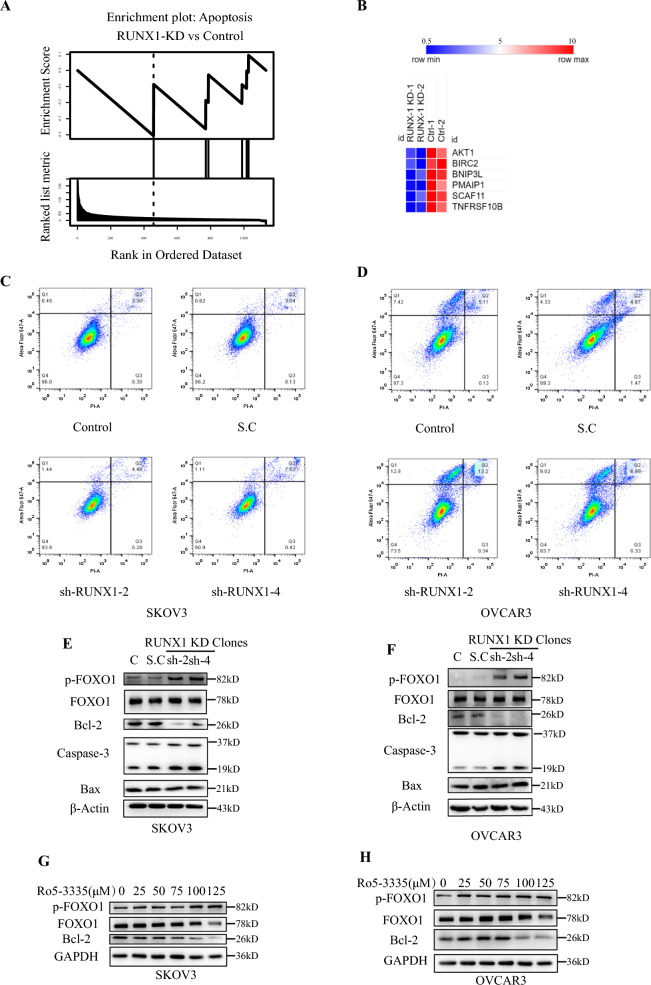
The RUNX1 knockdown impacted apoptosis in the ovarian cancer cell via the FOXO1-Bcl2 axis. GSEA identified the enrichment plot of apoptosis-related genes in RUNX1 KD compared to control cells (**A**). Heat maps significantly compare the gene expression involved in apoptosis regulation in RUNX1 KD and control groups (**B**). Annexin V-PI staining analysis by flow cytometry showed that the knockdown of RUNX1 enhanced the apoptosis level of ovarian cancer cells (**C** and **D**). Western blot detected apoptosis-related molecules in RUNX1 knockdown cell lines (**E** and **F**). Western blot detected of apoptosis-related molecules after Ro5-3335 treatment (**G** and **H**). *p < 0.05, **p < 0.01, and ***p < 0.001 compared to control cells expressing a scramble shRNA control, paired t-test

The apoptosis signaling pathway was extremely positive enriched pathways in the RUNX1 KD cells by GESA (Fig. 4A). These essential genes involved in apoptosis were significantly changed in the RUNX1 KD cell lines (Fig. 4B), including AKT1, BIRC2, BNIP3L, PMAIP1, SCAF11 and TNFRSF10B. Subsequently, flow cytometry analysis results indicated that the cell portion of apoptosis was significantly increased in RUNX1 knockdown ovarian cancer cell lines (SKOV3, OVCAR3) (Fig. 4C–D). In addition, the expression level of apoptosis-related key signalling molecules was impacted. Cleaved-caspase-3, which regulates apoptosis, was significantly upregulated in RUNX1 knockdown cells (Fig. 4E–F, Additional file 1: Fig. S3A–B). Meanwhile, the expression of anti-apoptotic protein Bcl-2 was significantly decreased (Fig. 4E–F, Additional file 1: Fig. S3A–B). However, RUNX1 knockdown did not affect the expression of the pro-apoptotic protein BAX (Fig. 4E–F, Additional file 1: Fig. S3A-B). Therefore, it is suggested that RUNX1 do not regulate apoptosis through the BAX/BAK pathway. Forkhead box O1 (FOXO1) is a transcription factor that regulates metabolism, cell proliferation, autophagy, and apoptosis. It is encoded by the *FKHR* gene [16, 17]. Meanwhile, studies have shown that FOXO1 plays a crucial role in apoptosis regulation [18–21]. In this study, the expression of FOXO1 was examined and the results showed that the level of p-FOXO1^Ser256^ was significantly increased in RUNX1 knockdown group, while the expression level of FOXO1 was almost unchanged (Fig. 4E–F, Additional file 1: Fig. S3A-B). Treated with Ro5-3335, a RUNX1-CBFβ interaction inhibitor, the level of p-FOXO1^Ser256^ and Bcl-2 were significantly changed in control cancer cell lines (SKOV3, OVCAR3), meanwhile the expression level of FOXO1 was almost unchanged after Ro5-3335 treatment (Fig. 4G–H, Additional file 1: Fig. S3C–D). These results imply that RUNX1 regulate apoptosis of ovarian cancer cells through FOXO1-Bcl2 axis, ultimately demonstrating its role in the regulation of ovarian cancer cellular functions.

### RUNX1 knockdown impaired EMT of cancer cells through the EGFR-AKT-STAT3 axis

**Fig. 5:**
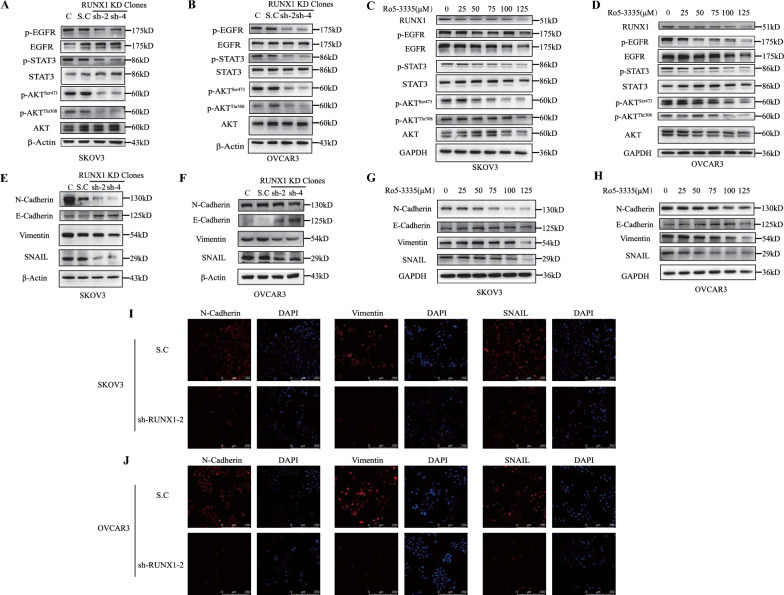
RUNX1 knockdown affects EMT in ovarian cancer cells via the EGFR-AKT-STAT3 axis. Western blot detected the signaling molecules change pattern in the RUNX1 KD cells that involved in the EMT related signaling pathway (**A** and **B)**. Western blot detected the signaling molecules change pattern after Ro5-3335 (a RUNX1 inhibitor) treatment that involved in the EMT related signaling pathway (**C** and **D**). Western blot detected of EMT-related molecules in RUNX1 knockdown cell lines (**E** and **F**). Western blot detected of EMT-related molecules after Ro5-3335 treatment (**G** and **H**). The expression of EMT-related molecules was analyzed by immunofluorescence in RUNX1 knockdown cell lines (**I** and **J**). *p < 0.05, **p < 0.01 and ***p < 0.001 as compared with control cells expressing a scramble shRNA control, paired t test

The VEGFA-VEGFR2 Signaling pathway was enriched in the RUNX1 knockdown ovarian cancer cells (Fig. 3C). It has been reported that RUNX1 regulated tumor function by activating downstream STAT3 and transcriptionally regulating EGFR gene expression [22]. In this study, RUNX1 knockdown significantly reduced the phosphorylation level of EGFR (Tyr1068), STAT3 (Tyr705), and AKT (Ser473, Thr308) in cancer cell lines (SKOV3, OVCAR3), and there were almost no significant differences in the expression levels of EGFR, STAT3 and AKT (Fig. 5A–B, Additional file 1: Fig. S4A–B). After treated with Ro5-3335, a RUNX1-CBFβ interaction inhibitor, the phosphorylation level of EGFR (Tyr1068), STAT3 (Tyr705) and AKT (Ser473, Thr308) were significantly decreased in control cancer cell lines (SKOV3, OVCAR3) and generated the similar pattern that RUNX1 knockdown induced (Fig. 5C–D, Fig. S4C–D). It was no significant difference in the expression levels of EGFR, STAT3 and AKT after Ro5-3335 treatment (Fig. 5C–D, Additional file 1: Fig. S4C–D). RUNX1 knockdown decreased EGFR activation, which affected STAT3 phosphorylation through AKT (Fig. 5A–B, Additional file 1: Fig. S4A–B), and achieved similar results by Ro5-3335 treatment (Fig. 5C–D, Additional file 1: Fig. S4C–D).

EGFR plays a key role in epithelial mesenchymal transformation (EMT). RUNX1 deletion can significantly reduce the expression of EGFR and downstream key factors. It is suggested that RUNX1 may regulate EMT through EGFR signalling pathway. The essential EMT-related proteins were significantly altered in RUNX1-KD cells lines (SKOV3, OVCAR3) (Fig. 5E–F, Additional file 1: Fig. S4E–F). Similar, Ro5-3335 treatment significantly altered expression level of RUNX1 and these EMT-related regulators (Fig. 5G–H, Additional file 1: Fig. S4G–H). In addition, the expression of the EMT essential protein (N-Cadherin, Vimentin, SNAIL) proteins were significantly altered after RUNX1 knockdown by immunofluorescence in RUNX1 knock-down cell lines (Fig. 5I–J).

WNT signaling pathway is an ancient and highly conserved pathway that modulates crucial of cell differentiation, cell migration and organogenesis [23, 24]. Many research works discovered the WNT signaling pathway plays a key role in multiple RUNX1-regulated cancers [25]. Wnt signaling has been reported to regulate EMT through β-catenin/SOX2 in colon cancer [26]. The Wnt signaling pathway was significantly positive enriched pathways in the RUNX1 KD cells (Additional file 1: Fig.S5A). The contribution of Wnt signaling in EMT was also investigated in RUNX1 knockdown cancer cells. RUNX1 knockdown did not affect β-catenin protein expression (Additional file 1: Fig. S5B–C). Therefore, the EMT changes induced by RUNX1 deletion may be regulated not by the classical Wnt signaling pathway but other signaling pathways. These results suggest that RUNX1 knockdown may affect ovarian cancer function by reducing EMT levels via the EGFR-AKT-STAT3 axis.

### RUNX1 Knockdown synergic with clinical chemotherapy drugs

**Fig. 6:**
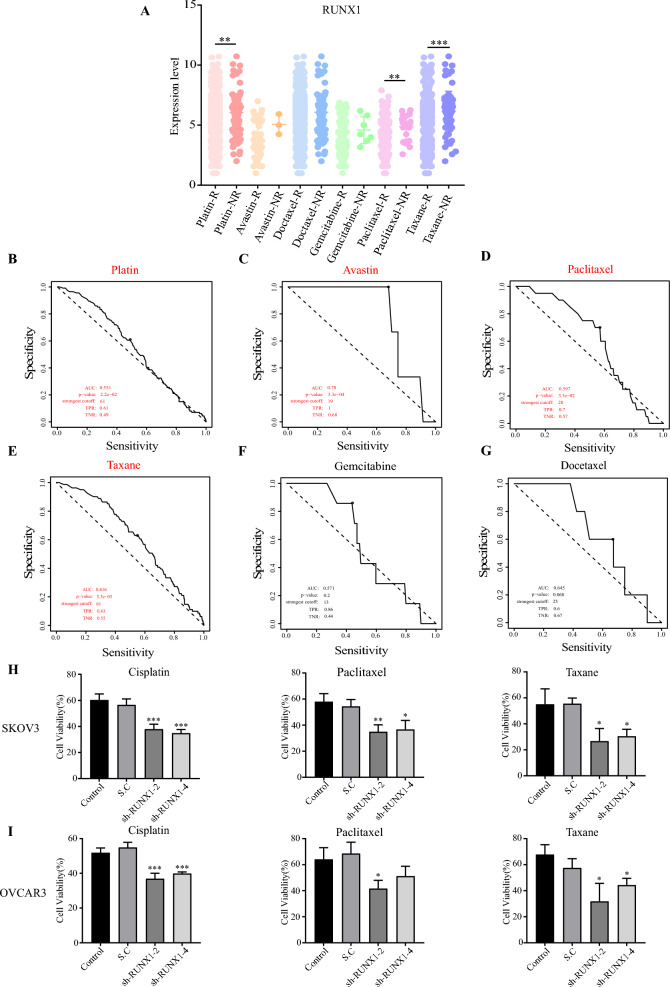
Correlation between six clinical drugs treated response and RUNX1 expression levels in ovarian cancer. RUNX1 expression levels in patients who responded to six clinical ovarian cancer agents versus patients who did not respond (**A**). Patients who responded to Avastin, paclitaxel, taxane, and platinum agents (cisplatin) had significantly lower levels of RUNX1 expression than those who did not respond. AUC and *p-value* of ROC of RUNX1 low expression level in treatment with clinical drugs (**B**, **C**, **D**, and **E**). There was no significant difference in RUNX1 expression between patients who responded to gemcitabine and docetaxel and those who did not reply (**F** and **G**). Effects on stably transfected sh-RUNX1 cell lines' proliferation after treatment with paclitaxel, taxane, and cisplatin (**H** and **I**). *p < 0.05, **p < 0.01 and ***p < 0.001 as compared with control cells expressing a scramble shRNA control, paired t test

Chemotherapy drugs (Paclitaxel, docetaxel, taxane, gemcitabine and platinum (carboplatin or cisplatin)) have been widely used as clinical treatment for ovarian cancer. However, patients will generate drug resistance after chemotherapy for a short period. It has been subsequently evaluated that the potential application of RUNX1 in clinical diagnosis and treatment and the relationship between RUNX1 expression and patient response to chemotherapy and clinical outcomes. Patients with lower RUNX1 expression lever are more likely to respond to chemotherapy agents (paclitaxel, taxane, and platinum agents (cisplatin) (Fig. 6A–G, Additional file 2: Table S2). In addition, chemotherapy drugs (paclitaxel, taxane, and platinum (cisplatin)) indicated significantly synergic anti-proliferation activity in RUNX1 knockdown SKOV3 cells (Fig. 6H) and OVCAR3 cells (Fig. 6I), respectively. Therefore, RUNX1 may provide a target in combination with the above clinical agents to treat HGSOC patients.

## Discussion

In this study, RUNX1 was identified as a potential key regulator involving in HGSOC development. TCGA and Kaplan Meier plotter databases showed that RUNX1 was highly expressed in ovarian cancer and significantly affected the survival curve. Patients with high RUNX1 expression demonstrated poorer prognosis, while genetic alternation of RUNX1 extended the survival time (Additional file 1: Fig. S1). In addition, RUNX1 knockdown impaired cellular functions of high grade serous ovarian cancer cell (Fig. 2C–G). This work opens a new unexplored avenue to investigate the enigmatic function of RUNX1 in neoplastic disease and identifies a novel role for the RUNX1 gene in the genetic landscape of ovarian cancer.

Apoptosis is essential for development and associated with many types of illness, including autoimmune diseases, neurodegenerative diseases, bacterial and viral diseases, heart diseases, and cancer progression [8, 27]. In this study, apoptosis pathway was enriched in RUNX1 KD cell lines. Previously, several researches also reported a close relationship between RUNX1 and apoptosis. Inhibition of RUNX1 promotes cisplatin-induced apoptosis in ovarian cancer cells [28]. The proapoptotic protein NOXA expression drives synthetic lethality to RUNX1 inhibition in pancreatic cancer [29]. RUNX1 inhibits proliferation and induces apoptosis via KLF4-mediated transactivation of P57 in leukemia cells [30]. Some microRNA’s influence on apoptosis is also realized by regulating RUNX1. MicroRNA-18-5p inhibits the oxidative stress and apoptosis of myocardium induced by hypoxia via targeting RUNX1 [31]. Knockdown of circ_0000512 inhibits cell proliferation and promotes apoptosis in colorectal cancer by regulating miR-296-5p/RUNX1 axis [32]. In this study, RUNX1 knockdown promoted apoptosis through the FOXO1-Bcl2 axis. FOXO1 regulates many targets, such as apoptosis and autophagy genes, antioxidant enzymes, cell cycle arrest genes, and metabolic and immune regulators [33, 34]. Loss of FOXO1 up-regulates human epidermal growth factor receptor 2(HER2)/neu expression and Wnt/β-catenin pathway, and then down-regulate pro-apoptotic genes [18, 19]. Studies in hepatocellular carcinoma (HCC) [35–37]. have shown that microRNA loci are frequently involved in tumor cell proliferation, migration, and invasion and contribute to direct inhibition and MDM2-mediated destabilization of FOXO1, suggesting that FOXO1 inhibition may be interested in HCC development. However, the role of FOXO1 in the development of ovarian cancer remains unclear. Interesting, it has not been reported that the specific mechanism by which RUNX1 regulates ovarian cancer cell apoptosis through FOXO1. In this study, RUNX1 deficiency promoted apoptosis through FOXO1-Bcl2 axis. The regulation of apoptosis by RUNX1 via FOXO1 was first reported. The mechanism by which RUNX1 mediates apoptosis was further elucidated. However, it is not clear how RUNX1 regulates FOXO1 to further affect Bcl2. Therefore, the pathway of FOXO1 mediated by RUNX1 will be explored in the future further study.

P53, as a transcription factor interacting with hundreds of genes, plays an important role in maintaining cellular homeostasis [38]. Recently, studies have shown that p53 can accumulate in the cytoplasm of HGSOC, further affecting its function [39]. Based on the near-universal presence of p53 mutations in HGSOC, surprisingly, there are currently no FDA-approved p53-based treatments for HGSOC [40]. The carcinogenic properties of RUNX1 in the absence of p53 were first presented in a study showing that the deletion of RUNX1 inhibited the development of T-cell lymphoma and extended their lifespan in p53-deficient mice [41]. At the same time, knockout of RUNX1 led to significant inhibition of adriamycin-mediated p53 acetylation at Lys-373/382, suggesting that RUNX1 plays a key role in Adriamycin-induced changes in P53 transcriptional activity [42]. These studies suggest that RUNX1 can act as an oncogene in the absence or mutation of p53. In this study, RUNX1 knockdown had similar effects on SKOV3 (p53 deficient cell line) function as OVCAR3. These suggest that RUNX1 may be a potential therapeutic target for patients with p53 deletion or mutation.

EGFR plays a crucial role in the growth, differentiation, and movement of normal and cancer cells [43]. EGFR could activate a variety of downstream pathways, such as the ERK, AKT and STAT3 pathways in ovarian cancer cells. VEGFR and EGFR share common downstream signaling pathways and may function exclusively of one another during oncogenesis and acquired therapeutic resistance can both trigger PI3K/AKT and RAS/RAF/ERK pathways. EGFR activation can drive HIF-1α up-regulation, leading to VEGF gene expression and providing a positive feedback loop in high grade serous ovarian cancer [44]. Interestingly, EGFR is over-expressed in up to 60% of ovarian epithelial malignancies, and its activation is associated with increased malignant tumor phenotype and poor patient prognosis [45, 46], while VEGF indicated lower positive expression in ovarian cancer patients (25.0%) [47]. In addition, RUNX1 has been reported to mediate tumor function by transcriptionally regulating EGFR expression, activating downstream STAT3 [22]. Targeting EGFR is attractive for cancer therapy due to its involvement in tumor initiation, angiogenesis, and metastasis. Combining these clues, we explored the EGFR-related signaling pathway, although the results revealed enrichment of the VEGFA-VEGFR2 signaling pathway (Fig. 3C). RUNX1 knockdown can significantly reduce phosphorylation level of EGFR (Tyr1068), STAT3 (Tyr705) and AKT (Ser473, Thr308), but there was no significant difference in the expression levels of EGFR, STAT3 and AKT. Therefore, decreased EGFR expression after RUNX1 knockdown can further lead to deactivated AKT. It is strongly suggested that overexpressed RUNX1 regulate the abnormal proliferation of ovarian cancer cells through the EGFR-AKT-STAT3 axis and RUNX1 work as a potential target for ovarian cancer treatment.

EMT is important for the development of many diseases, and the processes underpinning it are reactivated in fibrosis and cancer progression [48–51]. EGFR regulates EMT-related proteins via AKT and influences tumor cell function [52]. EGFR knockdown decreased the potential of migration and invasion of PCa cells with upregulation of E-Cadherin concomitant with the down-regulation of Vimentin and Snail [53]. Meanwhile, RUNX1 knockdown significantly decreased the phosphorylation level of EGFR and AKT in ovarian cancer cells. RUNX1 knockdown can significantly affect the expression of EMT-related proteins (Fig. 5E–F). These studies suggest that RUNX1 can regulate EMT through EGFR-AKT in ovarian cancer, thereby further regulating the invasion, migration, and metastasis of ovarian cancer.

Although taxane, paclitaxel and platinum agents (carboplatin or cisplatin) are widely used alone or in combination to treat ovarian cancer, chemotherapy resistance significantly reduces the efficacy of these agents [54]. Most patients treated with taxanes do not experience complete destruction of tumor cells. Moreover, resistance and relapse to taxanes are commonly observed, which eventually results in recurrence, poor prognosis, and significantly restricts the efficacy of taxane-based strategies for the treatment of ovarian cancer [55]. It is crucial to develop a new treatment plan as soon as possible. At present, the most promising research approach to tackle drug resistance involves the use of drug combinations. Ovarian cancer cells display a high level of RUNX1 expression, which activates the NF-κB pathway, leading to chemotherapy resistance. Inhibition of NF-κB has been shown to significantly decrease cell proliferation and promote apoptosis in drug-resistant ovarian cancer cells [56]. It has been suggested that the combination of chemotherapy drugs (taxane, paclitaxel, and platinum agents) with the deletion of RUNX1 gene can be more effective against chemoresistant ovarian cancer. This study found that reducing the expression of RUNX1 gene significantly increased the inhibitory effect of taxane, paclitaxel, carboplatin, or cisplatin on the proliferation of ovarian cancer cells. Therefore, ovarian cancer patients with RUNX1 genetic alternation, which has poor prognosis and drug resistance, have been treated with the combination of clinical chemotherapy drugs (cisplatin, taxane, paclitaxel, and platinum agents) as a novel therapy strategy may generate a significant therapeutic outcome. In the future, it is necessary and reasonable to investigate this proposal using patient-derived tumor cells and xenograft animal models. With the deepening of research, RUNX1 may become a potential biomarker for ovarian cancer patients or be identified as a potential target for clinical treatment and achieve better efficacy.

### Supplementary Information

**Additional file 1: Figure S1.** Overall survival and disease-free survival comparison of RUNX1 altered and unaltered patients in Ovarian Serous Cystadenocarcinoma via the cBioportal Genomics database. **Figure S2.** Two ovarian cancer cell lines with stable knockdown RUNX1 were established. **Figure S3.** The quantitative analysis of apoptosis-related molecules proteins after RUNX1 knockdown and Ro5-3335 treatment. **Figure S4.** The quantitative analysis of EMT-related signaling pathway proteins after RUNX1 knockdown and Ro5-3335 treatment. **Figure S5.** The effect of RUNX1 knockdown on the WNT signaling pathway. **Table S1.** ShRNA constructions and information for RUNX1.

**Additional file 2: Table S2.** Six clinical drugs treatment responses with or without RUNX1.

## Data Availability

This manuscript related data have supplied in the supporting materials.
