# Supplementary material for: RUNX1 knockdown induced apoptosis and impaired EMT in high-grade serous ovarian cancer cells
Source: J Transl Med. 2023 Dec 6;21:886. doi: 10.1186/s12967-023-04762-8 (PMC10702124; doi:10.1186/s12967-023-04762-8)
Supplement: Supplementary file 1 — Additional file 1: Figure S1. Overall survival and disease-free survival comparison of RUNX1 altered and unaltered patients in Ovarian Serous Cystadenocarcinoma via the cBioportal Genomics database. Figure S2. Two ovarian cancer cell lines with stable knockdown RUNX1 were established. Figure S3. The quantitative analysis of apoptosis-related molecules proteins after RUNX1 knockdown and Ro5-3335 treatment. Figure S4. The quantitative analysis of EMT-related signaling pathway proteins after RUNX1 knockdown and Ro5-3335 treatment. Figure S5. The effect of RUNX1 knockdown on the WNT signaling pathway. Table S1. ShRNA constructions and information for RUNX1. [file 12967_2023_4762_MOESM1_ESM.pdf]

RUNX1 knockdown induced apoptosis and impaired EMT in high-grade serous ovarian cancer cells

Yuanzhi Chen[1,2], Zhicheng He[1,2], Shuting Yang[1,4], Cheng Chen[1,2], Wenyong Xiong[1], YingYing He[3,*], Shubai Liu[1,2, *]

[1] State Key Laboratory of Phytochemistry and Plant Resources in West China, Kunming Institute of Botany, Chinese Academy of Sciences, Kunming, 650201 Yunnan, P. R. China.

[2] University of Chinese Academy of Sciences, Beijing 100049, China.

[3] School of Chemical Science & Technology, Yunnan University, Kunming, Yunnan 650091, China.

[4] School of Life Science, Yunnan University, Kunming, China.

***Correspondence:**

Dr. Shubai Liu

State Key Laboratory of Phytochemistry and Plant Resources in West China, Kunming Institute of Botany, the Chinese Academy of Sciences, #132 Lanhei Road, Panlong District, Kunming, Yunnan 650201, China. Tel: 86-871-6522 3309

E-mail: liushubai@mail.kib.ac.cn

or

Dr. YingYing, He

School of Chemical Science & Technology, Yunnan University, Kunming, Yunnan 650091, China.

E-mail: Yingying.he10@gmail.com

Additional file 1: Figure & Table Legends:

Figure.S1. Overall Survival and Disease-Free Survival comparison of RUNX1 altered and unaltered patients in Ovarian Serous Cystadenocarcinoma via the cBioportal Genomics database. Genetically altered of RUNX1 gene occurs in ovarian cancer patients (A). Overall Survival comparison of RUNX1 altered patients and unaltered patients (B). Disease-Free Survival comparison of RUNX1 altered patients and unaltered patients (C).

Figure.S2. Two ovarian cancer cell lines with stable knockdown RUNX1 were established. Western blot confirmed RUNX1 stable knockdown in ovarian cancer cells (SKOV3 and OVCAR3) constructed by plasmids containing RUNX1-targeting shRNA (A and B).

Figure.S3. The quantitative analysis of apoptosis-related molecules proteins after RUNX1 knockdown and Ro5-3335 treatment. The quantitively analysis of the apoptosis-related molecules in RUNX1 knockdown cell lines (A and B). The quantitative analysis of the apoptosis-related molecules after Ro5-3335 (a RUNX1 inhibitor) treatment (C and D). * $p < 0.05$, ** $p < 0.01$, and *** $p < 0.001$ compared to control cells expressing a scramble shRNA control, paired t-test.

Additional file 1: Figure 4. The quantitative analysis of EMT-related signaling pathway proteins after RUNX1 knockdown and Ro5-3335 treatment. The

quantitative analysis of the signaling molecules changes pattern in the RUNX1 KD cells involved in the EMT-related signaling pathway (A&B). The quantitative analysis of the signaling molecules changes pattern after Ro5-3335 (a RUNX1 inhibitor) treatment involved in the EMT-related signaling pathway (C and D). The quantitively analysis of EMT-related molecules in RUNX1 knockdown cell lines (E and F). The quantitative analysis of EMT-related molecules after Ro5-3335 treatment (G and H). * $p < 0.05$, ** $p < 0.01$ and *** $p < 0.001$ compared to control cells expressing a scramble shRNA control, paired t-test.

Additional file 1: Figure 5. The effect of RUNX1 knockdown on the WNT signaling pathway. GSEA identified the enrichment plot of WNT signaling-related genes in RUNX1 KD compared to control cells (A). Western blot detected the critical molecule of WNT signaling in RUNX1 knockdown cell lines (B and C).

Table S1. ShRNA constructions and information for RUNX1.

Table S2. Six clinical drug treatment responses with or without RUNX1.

A

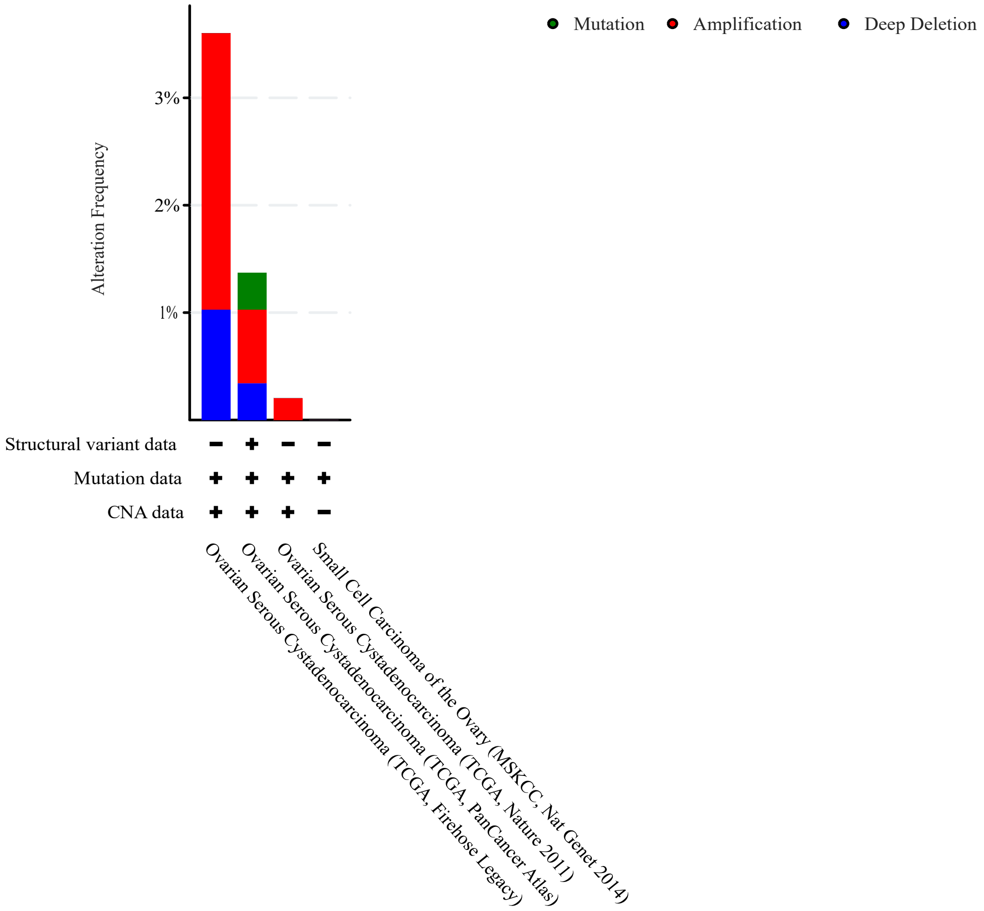
Mutation
Amplification
Deep Deletion
Alteration Frequency
3%
2%
1%
Structural variant data
Mutation data
CNA data
Ovarian Serous Cystadenocarcinoma (TCGA, Firehose Legacy)
Ovarian Serous Cystadenocarcinoma (TCGA, PanCancer Atlas)
Ovarian Serous Cystadenocarcinoma (TCGA, Nature 2011)
Small Cell Carcinoma of the Ovary (MSKCC, Nat Genet 2014)

B

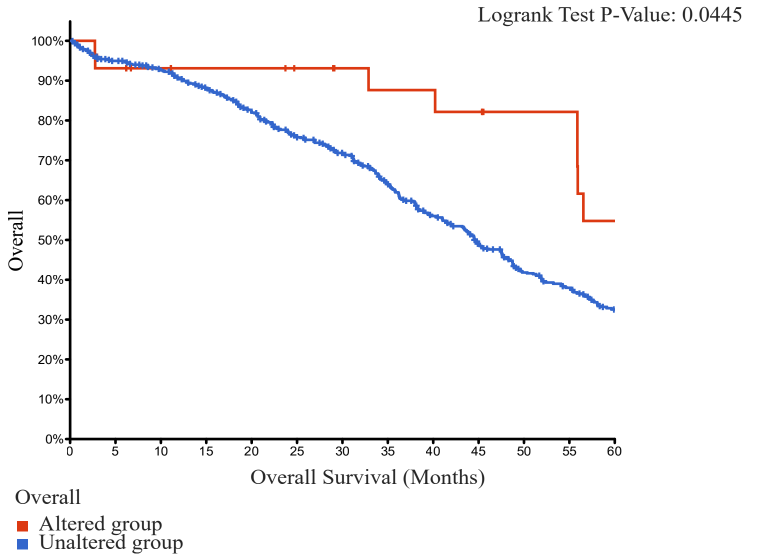
Logrank Test P-Value: 0.0445
Overall
Overall Survival (Months)
Overall
Altered group
Unaltered group

C

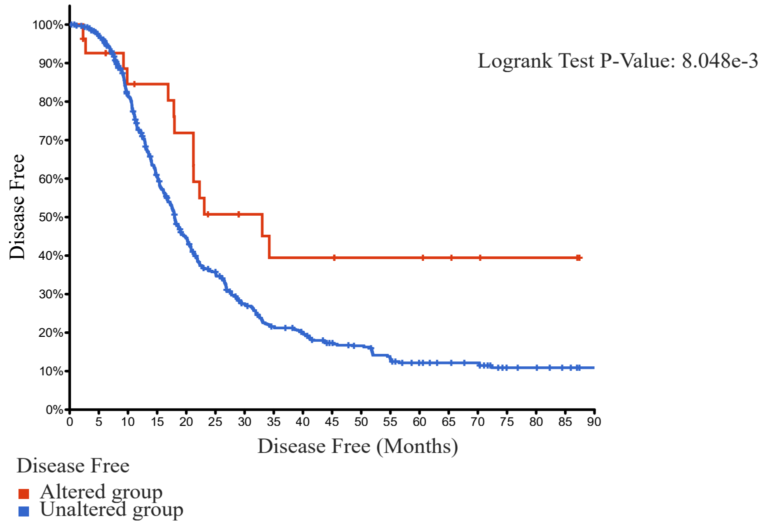
Logrank Test P-Value: 8.048e-3
Disease Free
Disease Free (Months)
Disease Free
Altered group
Unaltered group

A

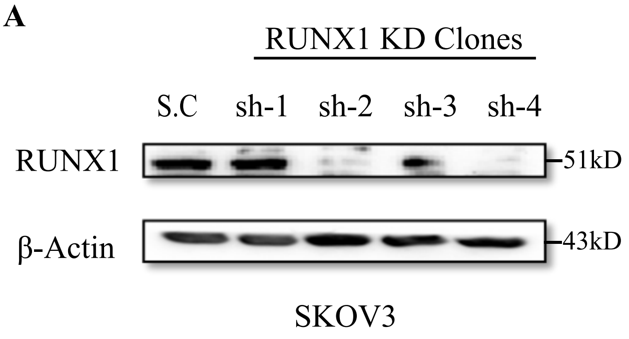
RUNX1 KD Clones
S.C
sh-1
sh-2
sh-3
sh-4
RUNX1
51kD
β-Actin
43kD
SKOV3

B

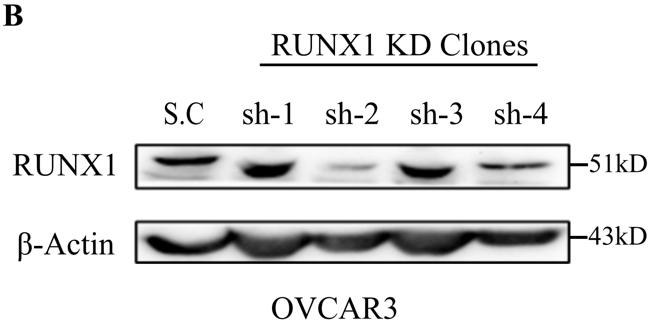
RUNX1 KD Clones
S.C
sh-1
sh-2
sh-3
sh-4
RUNX1
51kD
β-Actin
43kD
OVCAR3

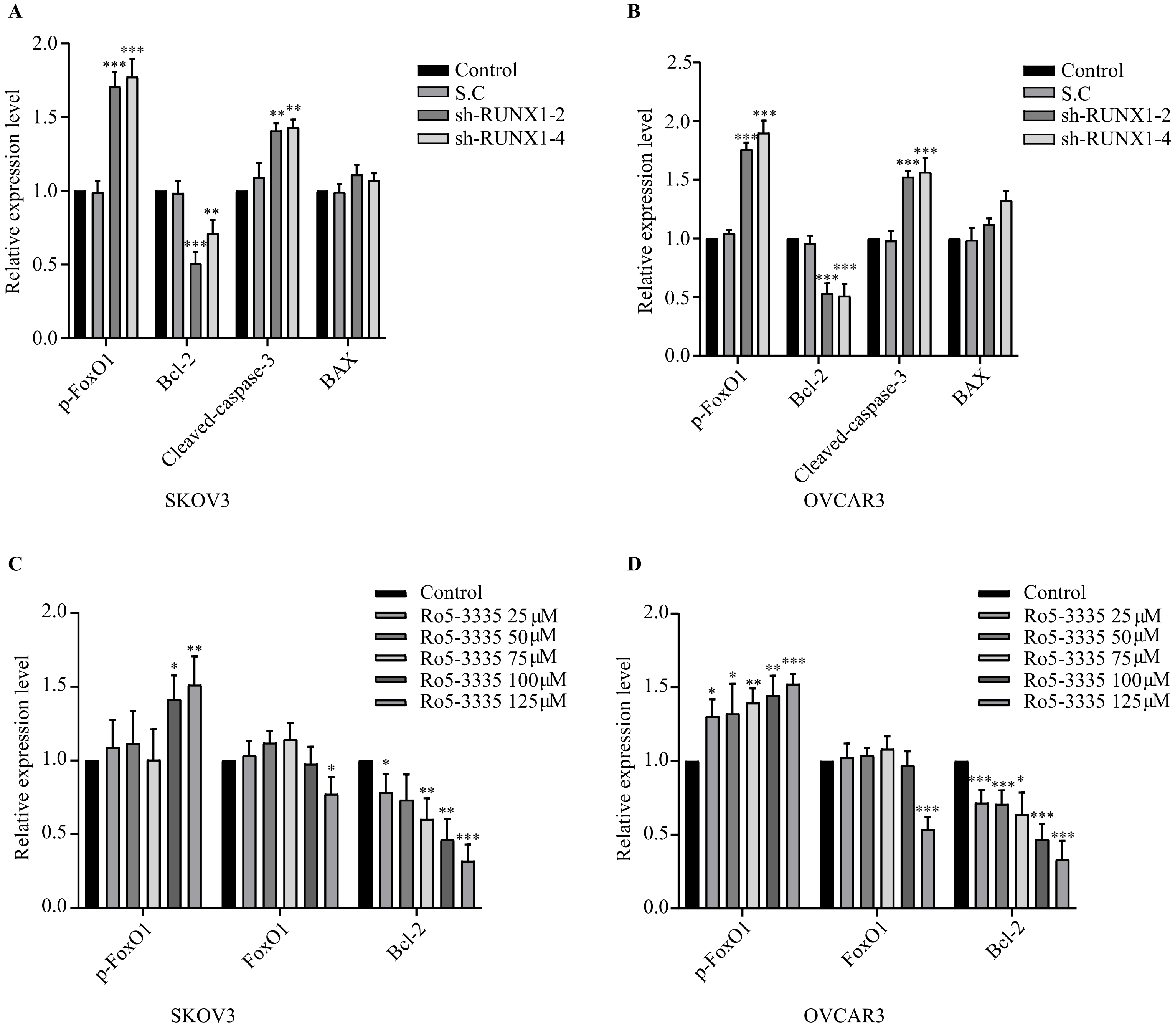

A
B
C
D
Relative expression level
Control
S.C
sh-RUNX1-2
sh-RUNX1-4
p-FoxO1
Bcl-2
Cleaved-caspase-3
BAX
SKOV3
OVCAR3
Ro5-3335 25 μM
Ro5-3335 50μM
Ro5-3335 75μM
Ro5-3335 100μM
Ro5-3335 125μM
FoxO1

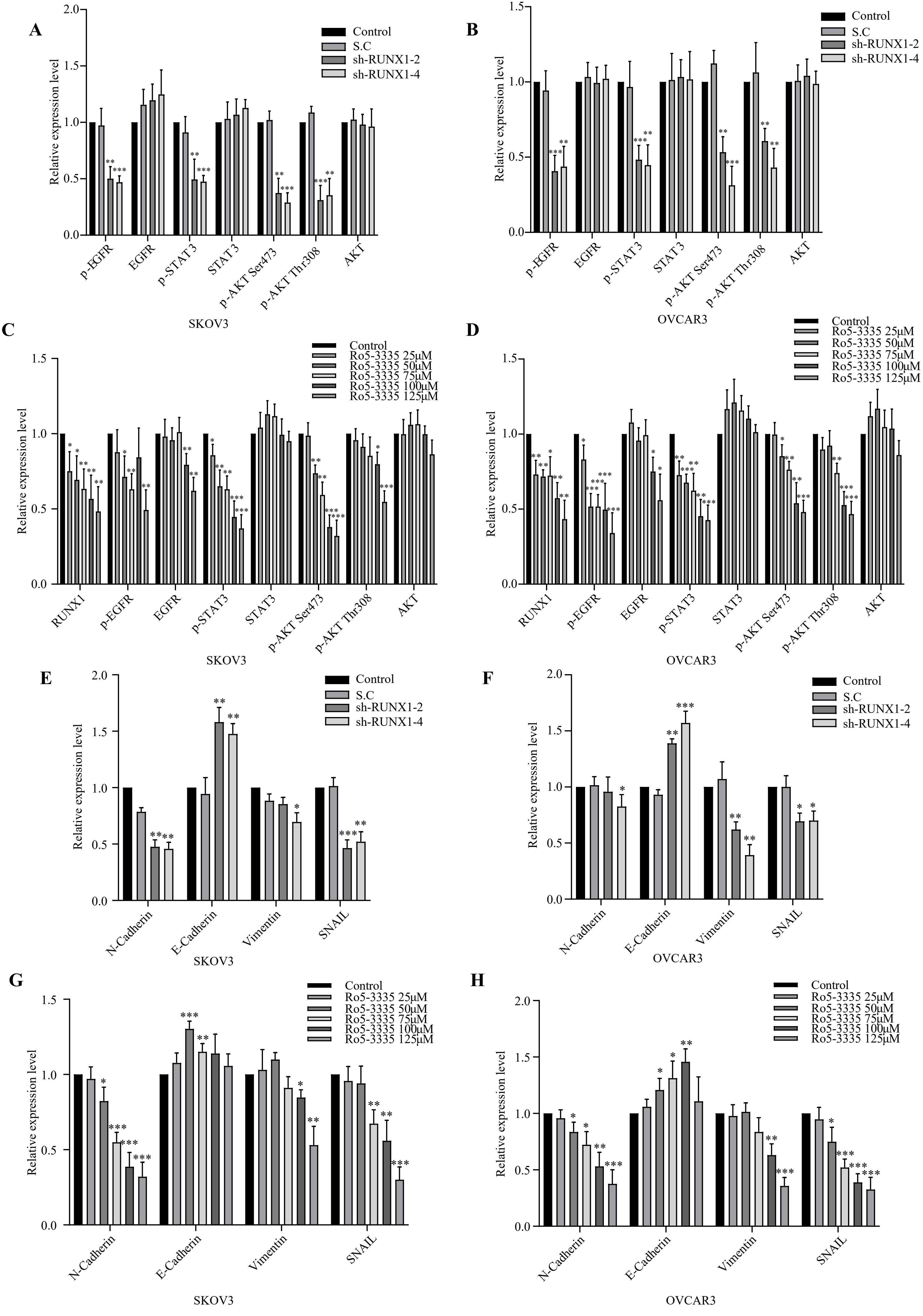
A
Control
S.C
sh-RUNX1-2
sh-RUNX1-4
Relative expression level
p-EGFR
EGFR
p-STAT3
STAT3
p-AKT Ser473
p-AKT Thr308
AKT
SKOV3
B
OVCAR3
C
Ro5-3335 25μM
Ro5-3335 50μM
Ro5-3335 75μM
Ro5-3335 100μM
Ro5-3335 125μM
RUNX1
D
E
N-Cadherin
E-Cadherin
Vimentin
SNAIL
F
G
H

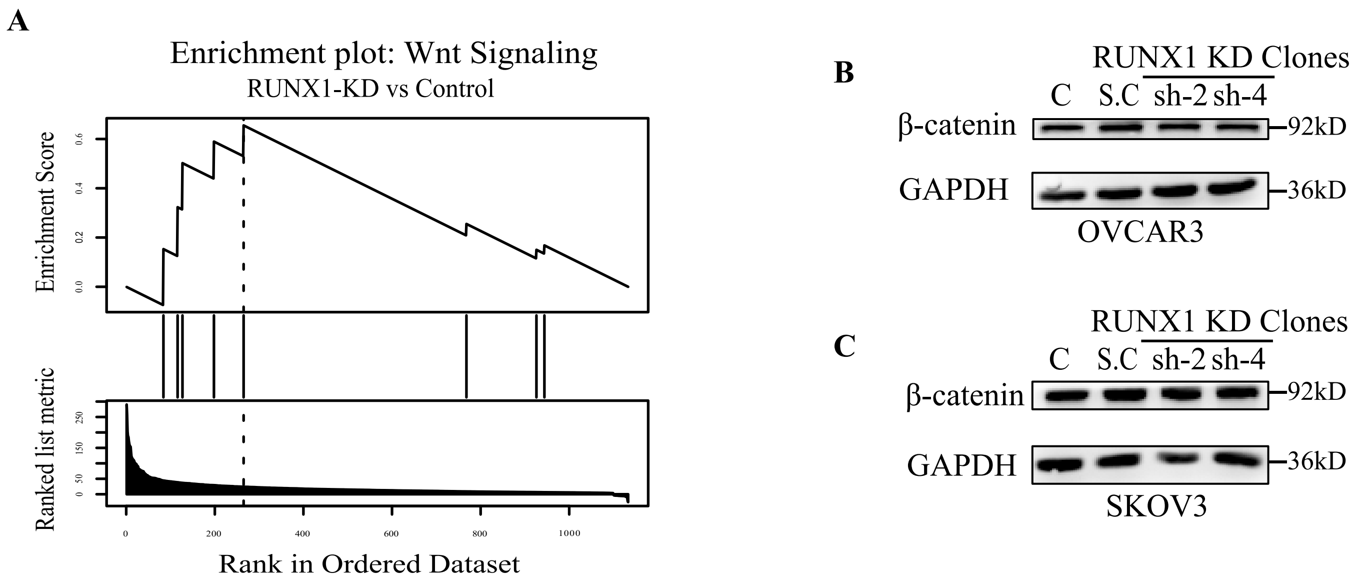
A
Enrichment plot: Wnt Signaling
RUNX1-KD vs Control
Enrichment Score
Ranked list metric
Rank in Ordered Dataset
B
RUNX1 KD Clones
C
S.C
sh-2
sh-4
β-catenin
92kD
GAPDH
36kD
OVCAR3
C
RUNX1 KD Clones
C
S.C
sh-2
sh-4
β-catenin
92kD
GAPDH
36kD
SKOV3

Table.S1 ShRNA constructions and information for RUNX1.

Gene name	TRC numbers	shRNA sequnces
RUNX1-shRNA-1	TRCN0000338428	CCGGCTACGATCAGTCCTACCAATACTCGAGTATTGGTAGGACTGATCGTAGTTTTTT
RUNX1-shRNA-2	TRCN0000338490	CCGGGAACCACTCCACTGCCTTTAACTCGAGTTAAAGGCAGTGGAGTGGTTCTTTTTT
RUNX1-shRNA-3	TRCN0000338427	CCGGGAACCAGGTTGCAAGATTTAACTCGAGTTAAATCTTGCAACCTGGTTCTTTTTT
RUNX1-shRNA-4	TRCN0000013658	CCGGGCCTTGAAATACCTGTTTCTTCTCGAGAAGAAACAGGTATTTCAAGGCTTTTTT
